# Effects of a Honeybee Sting on the Serum Free Amino Acid Profile in Humans

**DOI:** 10.1371/journal.pone.0103533

**Published:** 2014-07-29

**Authors:** Jan Matysiak, Paweł Dereziński, Agnieszka Klupczyńska, Joanna Matysiak, Elżbieta Kaczmarek, Zenon J. Kokot

**Affiliations:** 1 Department of Inorganic & Analytical Chemistry, Poznan University of Medical Sciences, Poznań, Poland; 2 Ward of Paediatric Diseases, L. Perzyna Regional Unified Hospital in Kalisz, Kalisz, Poland; 3 Department of Bioinformatics and Computational Biology, Poznan University of Medical Sciences, Poznań, Poland; Rosalind Franklin University, United States of America

## Abstract

The aim of this study was to assess the response to a honeybee venom by analyzing serum levels of 34 free amino acids. Another goal of this study was to apply complex analytic-bioinformatic-clinical strategy based on up-to-date achievements of mass spectrometry in metabolomic profiling. The amino acid profiles were determined using hybrid triple quadrupole/linear ion trap mass spectrometer coupled with a liquid chromatography instrument. Serum samples were collected from 27 beekeepers within 3 hours after they were stung and after a minimum of 6 weeks following the last sting. The differences in amino acid profiles were evaluated using MetaboAnalyst and ROCCET web portals. Chemometric tests showed statistically significant differences in the levels of L-glutamine (Gln), L-glutamic acid (Glu), L-methionine (Met) and 3-methyl-L-histidine (3MHis) between the two analyzed groups of serum samples. Gln and Glu appeared to be the most important metabolites for distinguishing the beekeepers tested shortly after a bee sting from those tested at least 6 weeks later. The role of some amino acids in the response of an organism to the honeybee sting was also discussed. This study indicated that proposed methodology may allow to identify the individuals just after the sting and those who were stung at least 6 weeks earlier. The results we obtained will contribute to better understanding of the human body response to the honeybee sting.

## Introduction

Stinging by insects of *Hymenoptera* order is an important problem in allergological practice [1], [2]. However, the mechanisms of the organism response to stings are still not fully explained. Being regularly exposed to the honeybee venom allergens, the beekeepers are a very convenient population to study the immune response directly after a sting [3] or to investigate the changes in the profile of endogenous compounds, including amino acids (AAs). The results of many metabolomic experiments broaden the knowledge about complex responses of organisms to pathophysiological stimuli, which in turn may contribute to the development of new diagnostic strategies [4], [5]. It was also suggested that AAs may inhibit IgE binding to allergens [6]. Thus, the determination of AA concentration profile in the body fluids may provide a weakly invasive diagnostic tool in the allergic diseases [7].

Amino acids play essential roles as key components of peptides, proteins and phospholipids. They can also serve as: precursors (biogenic amines, glucose, heme), neurotransmitters and donors of amine group. It is known that changes in amino acids availability have profound effects on many aspects of cellular functions, including a regulation of cell signaling, gene expression and the transportation of amino acids themselves [8].

Free amino acid profiles are influenced by metabolic variations that reflect the physiological or pathological condition of an organism [9]. Their serum levels in individuals alter in various conditions, including liver failure [10], [11], renal failure [12], cancer [13]–[17], diabetes [18], or gastrointestinal disorders [19]. It is suggested that AA profiles may provide information on metabolic disturbances or distinct metabolism. Changed amino acid profile may not only refer to increase or decrease in the concentration of individual amino acids in the body fluids but is associated with specific groups of AAs, such as: branched chain amino acids or aromatic amino acids.

Mass spectrometry-based serum metabolite profiling is a promising technique to analyze complex metabolic alterations. This may broaden the pathophysiological understanding of an organism’s response to a honeybee sting and may function as additional information in the honeybee venom allergy diagnosis. Several methods have been devised for the determination of amino acids in the human body fluids. They include capillary electrophoresis, high performance liquid chromatography, thin layer chromatography, and ion exchange chromatography [20]–[24]. The most popular method of amino acid analysis in the body fluids involves ion exchange chromatography with the post-column derivatization, taking advantage of ninhydrin reaction. A disadvantage of the technique involves its low throughput (analysis of a single sample takes 2.5 h). In the recent years, due to the use of liquid chromatography coupled to tandem mass spectrometry (LC-MS/MS), a potential for the development of a new rapid technique for quantitative analysis of amino acids in the body fluids has appeared [25].

There is virtually no literature studying the effects of a bee sting at the metabolomic level. Therefore, the aim of this pilot study was to assess the response to the honeybee venom in the beekeepers by analyzing their serum levels of 34 free AAs. Another goal of this study was to apply complex analytic-bioinformatic-clinical strategy based on up-to-date achievements of mass spectrometry in metabolomic profiling. This is the first report in the available literature on clinical studies that is focused on the characterization of serum AAs profiles after a sting.

## Materials and Methods

### Study participants and serum samples

The study participants were volunteers who were recruited at the meetings of beekeeping associations. Serum samples were collected twice from 27 beekeepers: first time within 3 hours after a bee sting (stung beekeepers) and second time after at least 6 weeks following the last bee sting (after minimum 6 weeks from the end of the beekeeping season – non-stung beekeepers). In order to reduce the diet induced differences in the free amino acids concentrations, the samples were collected after overnight fasting. The applied approach in the selection of the study group and the control group contributes to eliminate possible differences in serum free amino acid concentrations caused by gender, age, ethnic origin, disease, and lifestyle factors (physical activity, smoking, alcohol consumption, drugs). The beekeepers’ age range was 20 to 80 years. The characteristics of the beekeepers involved in the study were prepared based on a questionnaire (Table 1).

**Table 1:** Characteristics of the beekeepers involved in the study (n = 27).

Characteristic		N	%
**Age [years]**	21 to 40	8	29.6
	41 to 60	8	29.6
	Above 61	11	40.8
**Sex**	male	24	88.9
	female	3	11.1
**The number of colonies in the apiary**	1 to 20	5	18.5
	21 to 40	16	59.2
	more than 40	6	22.2
**Length of time in the apiary [years]**	1 to 10	10	37.0
	11 to 20	9	33.3
	more than 20	8	29.7
**The number of stings received daily**	less than 1	15	55.6
	1 to 5	4	14.8
	6 to 10	4	14.8
	more than 10	4	14.8
**The number of days working in the apiary per week**	1 to 2	14	51.9
	3 to 4	9	33.3
	5 to 6	4	14.8
**Using of protective clothing**	Some elements	13	48.1
	The whole outfit	14	51.9

All the samples were stored at –80°C until the analysis and analyzed within 1 analytical cycle. The study was conducted with the approval of the Bioethics Committee of the Poznan University of Medical Sciences, Poland (Resolution No. 324/11) and fulfilled the requirements of the Helsinki declaration. All beekeepers gave their written consent to participate in the study.

In order to describe the overall patterns of change or latent structure in the data, critical statistical evaluation was applied.

### Chemicals

Both aTRAQ kit for the analysis of amino acids in the physiological fluids and the Control Plasma were purchased from AB Sciex (Framingham, MA, USA). The kit comprises all reagents necessary for the amino acids labeling with the 121 aTRAQ tag, a solution of amino acids labeled with the 113 aTRAQ tag used as internal standards and mobile phase modifiers (formic acid and heptafluorobutyric acid). HPLC-grade methanol for LC–MS was supplied by J.T. Baker (Griesheim, Germany). Deionized water from Millipore Simplicity UV water purification system (Waters Corporation, Milford, MA, USA) was used.

### aTRAQ methodology

The aTRAQ kit for amino acids analysis enables the quantification of 42 amino acids from a range of physiological fluids and matrices, i.e. serum, plasma, urine, cerebrospinal fluid and tissue extracts. It contains aTRAQ Reagent Δ8 for labeling amino acids in the samples with the 121 aTRAQ tag. The reagent tags the primary and secondary amino groups of amino acids. It contains a reactive amino group and a reporter ion. The kit also contains a mixture of 44 Δ0-labeled amino acids with the 113 aTRAQ tag to be used as internal standards (2 of them serve as internal standards for norleucine and norvaline). The reporter ion of the 121 aTRAQ tag has a mass to charge ratio (m/z) of 121, whereas the reporter ion of the 113 aTRAQ tag has m/z of 113. Therefore, amino acids present in the physiological samples and internal standards, labeled with two different tags, are 8 mass units apart. They have the same retention times, but they can be distinguished due to the unique mass transitions.

After preparation, the samples were analyzed with the LC-MS/MS method using a triple quadrupole system operating in the scheduled multiple reaction monitoring (sMRM) mode. The first quadrupole (Q1) was set to select precursor ions, which were Δ8-labeled amino acids originating from the physiological samples and Δ0-labeled amino acids (internal standards). The precursor ions were then fragmented in the second quadrupole (Q2), which served as a collision cell. The third quadrupole (Q3) selected product ions for detection. They were the cleaved reporter ions of m/z values of 113 and 121. The use of the scheduled MRM mode has a number of advantages, especially when a large number of MRM transitions are monitored. The sMRM mode sets a window around the retention time during which each amino acid and internal standard is monitored, thus decreasing the number of MRM transitions monitored within a given time window. That allows for maximizing the dwell time and collecting more data points per peak. The benefits involve better data quality and reproducibility resulting in more accurate quantitation. The chromatogram for each mass transition was plotted and the areas of integrated chromatographic peaks were used for quantification of amino acids. Concentrations of the compounds were determined by dividing the Δ8-labeled amino acid peak area by the peak area of its corresponding Δ0-labeled internal standard and multiplying by the concentration of the internal standard in a sample.

The benefits arising from using aTRAQ reagents with LC-MS/MS system have been confirmed in the literature data [25]. It has been proven that applying aTRAQ reagents for the quantitative analysis of amino acids in the biological fluids increases the specificity of the method and significantly reduces the duration of the analysis (one analytical run takes 18 minutes).

### Sample preparation for AAs analysis

The first step of the sample preparation procedure was the precipitation of any proteins from the specimens by adding 10 µl of sulfosalicylic acid to 40 µl of the serum sample. The sample was mixed and centrifuged at 10 000×g for 2 minutes. Ten microliters of the supernatant were diluted with 40 µl of the borate buffer that provided alkaline pH necessary for the labeling reaction, then vortexed and centrifuged. An aliquot of 10 µl of the supernatant/borate buffer mixture was labeled with 5 µl of the previously diluted reagent solution (aTRAQ Reagent Δ8), vortexed and centrifuged. The sample was incubated at room temperature for at least 30 minutes. In order to stop the labeling reaction, 5 µl of hydroxylamine was added. After mixing and centrifuging, the sample was incubated at room temperature for at least 15 minutes. Then 32 µl of the previously prepared internal standard solution was added to the sample, the aliquot was mixed and centrifuged. The sample volume was reduced by evaporating in a vacuum concentrator for 15 minutes. Then 20 µl of water were added to the sample. After mixing the sample was transferred to an autosampler vial.

In order to check labeling efficiency, the non-proteogenic amino acids (norleucine and norvaline) were used.

### LC-ESI-QqQ-MS/MS

The analyses were performed using 4000 QTRAP mass spectrometer (AB Sciex, Framingham, MA, USA) coupled with LC instrument 1260 Infinity (Agilent Technologies, Santa Clara, CA, USA). The amino acids were separated on AB Sciex C18 (5 µm, 4.6 mm×150 mm) column by binary gradient of water (mobile phase A) and methanol (mobile phase B), both containing 0.1% formic acid and 0.01% heptafluorobutyric acid. The flow rate of mobile phase was maintained at 0.8 ml/min. The gradient elution was: 0 min, 2% B; 0–6 min linear from 2 to 40% B; 6–10 min, 40% B; 10–11 min linear from 40 to 90% B; 11–12 min, 90% B; 12–13 min linear from 90 to 2% B; 13–18 min, 2% B. The separation temperature was set at 50°C.

The 4000 QTRAP mass spectrometer equipped with an electrospray ionization (ESI) source was used. The analyses were carried out in a positive ionization mode with ion spray voltage 4500 V, entrance potential 10 V, declustering potential 30 V and collision cell exit potential 50 V. The temperature, ion source gas 1 and ion source gas 2, were set at 600°C, 60 psig and 50 psig, respectively. For amino acid fragmentation the collision energy of 30 V was applied, except from argininosuccinic acid (Asa), L-ornithine (Orn), cystathionine (Cth), L-cystine (Cys), δ-hydroxylysine (Hyl), L-lysine (Lys) and L-homocystine (Hcy) (50 V). Nitrogen was used as a curtain gas (20 psig) and a collision gas (medium). Scheduled multiple reaction monitoring (sMRM) mode was used. Data acquisition and processing were controlled using Analyst 1.5 software (AB Sciex).

### Validation of LC-ESI-QqQ-MS/MS method

The applied method was characterized by the following validation parameters: limit of quantitation, linear dynamic range, precision and accuracy. In order to verify the validation of the method, the following parameters were assessed: accuracy, intra-day and inter-day precision of amino acid concentrations and their retention times. Additionally, retention time stability and sensitivity of the LC-MS system were checked.

Accuracy of the method was proved by analyzing the Control Plasma with reference ranges of 25 amino acids concentrations. The Control Plasma was analyzed with each new set of reagents (one reagent kit allows to analyze 50 samples). For the estimation of the intra-day precision one serum sample was prepared three times simultaneously and measured in three replicates, resulting in nine analyses of that sample. For the estimation of the inter-day precision, the same procedure as for the intra-day precision was carried out for three consecutive days. As a result, the total amount of analyses of the sample for inter-day precision was nine.

Every single day before running the samples, the system suitability test (SST) was performed. SST warmed up the entire LC-MS system and verified that it was working properly. The test also checked the retention times stability and sensitivity levels for the instrument. A positive result of the SST was the condition for performing further analyses of the samples.

The presence of an internal standard for each amino acid compensates matrix effects and interferences from the eluents used during the analysis. Moreover, it improves the precision and accuracy of quantitations. In addition, the availability of an internal standard for each analyte improves the specificity of the method. Confirmation of amino acid identification is based on unique mass transitions for each analyte and agreement of retention times between internal standards and amino acids in the physiological samples.

### Data processing and statistical analysis

Chemometric analyses including t-tests, Partial-Least Squares Discriminant Analysis (PLS-DA) and Significance Analysis of Microarray (SAM) were performed with the MetaboAnalyst 2.0 web portal (www.metaboanalyst.ca) [26]. ROC (receiver operating characteristic) Curves Analysis was performed using ROCCET web portal (www.roccet.ca) [27]. To reduce systematic variance and to improve the performance for downstream statistical analysis normalization and transformation of raw data were performed before the t-tests, PLS-DA and SAM analysis. Normalization by sum was used to overcome the variance between the analyzed samples. To make each feature comparable in magnitude to each other the data were transformed by taking the natural log of the concentration values of the analyzed amino acids. The data were additionally auto-scaled (mean-centered and divided by the standard deviation of each variable). These transformations enabled us to obtain normal distribution of variables that allowed applying parametric tests in the data evaluation, which was confirmed by Shapiro-Wilk test.

Univariate analysis was used to check the differences in the concentrations of the analyzed amino acids between the stung and non-stung beekeepers. Paired t-test was applied to examine each variable (serum amino acid concentration) separately, without taking into account the effect of multiple comparisons.

PLS-DA and SAM were used both for the classification and significant feature selection. A variable importance in projection (VIP) plot, which is commonly used in PLS-DA, is ranking the amino acids based on their importance in discrimination between the stung and non-stung beekeepers. VIP score is a weighted sum of squares of the PLS loadings. The amount of explained Y-variance in each dimension influenced the weights [28]. SAM can help to dissolve the problems with False Discovery Rate (FDR), when running multiple analyses on high-dimensional data.

ROC Curves Analysis was used to determine a cut-off value of differentiating factors (serum concentrations of amino acids) for two analyzed groups of beekeepers. In a ROC curve, the true positive rate (sensitivity) is plotted against the false positive rate (1-specificity) for different cut-off points of a given parameter. The test, which can perfectly distinguish between two diagnostic groups, has a ROC curve that passes through the upper left corner of the plot (100% sensitivity, 100% specificity). Thus, the closer the ROC curve is to the upper left corner, the better diagnostic power of the test is achieved. In turn, the closer the ROC curve to the diagonal line, the poorer accuracy of the test. ROC curve is using non-parametric algorithms so there is no need to perform data normalization.

In all analyses, the level of p below 0.050 was taken as indicating statistical significance. Normal distribution of the analyzed data was tested using the Shapiro-Wilk test.

## Results and Discussion

The aim of this study was to assess the response of the beekeepers to the honeybee venom by analyzing serum levels of 34 free amino acids. Amino acid profiles were determined using hybrid triple quadrupole/linear ion trap mass spectrometer coupled with liquid chromatography instrument. Serum samples were collected from 27 beekeepers within 3 hours after a sting and after a minimum of 6 weeks following the last sting.

**Figure 1:**
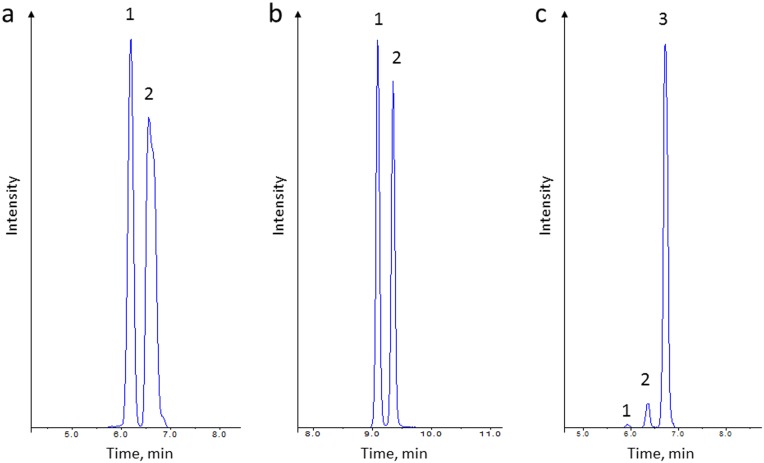
Extracted ion chromatograms of isobaric amino acids acquired during the analysis of one of the serum sample. **a:** identified amino acids: 1-1MHis; 2-3MHis; **b:** identified amino acids: 1-Val; 2–Nval; **c:** identified amino acids: 1-Sar; 2-bAla; 3–Ala.

Concentrations of the amino acids in the analyzed serum of the beekeepers are presented in Table 2. Csv file with raw data is included in File S1. Despite applying LC-ESI-QqQ-MS/MS method that allows the determination of 42 amino acids in a single run, only 34 amino acids were detected in the analyzed samples. The following amino acids were not detected in the samples analyzed with this methodology: O-phospho-L-serine (PSer), O-phosphoethanolamine (PEtN), argininosuccinic acid (Asa), L-homocitrulline (Hcit), L-anserine (Ans), L-carnosine (Car), cystathionine (Cth) and L-homocystine (Hcy). In most cases, a chromatographic resolution of analytes is not necessary due to using a mass spectrometer mode. However, a chromatographic separation is obligatory in the case of isobaric amino acids and their corresponding internal standards because of the same mass transitions, which is the characteristic feature of isobaric amino acids (Figure 1). According to the Human Metabolome Database (www.hmdb.ca), none of the amino acid concentrations analyzed in the group of the beekeepers exceeded the thresholds for healthy people, which provides strong evidence, that the volunteers involved in this study did not suffer from any disease likely to interfere with the results obtained. Moreover, in order to assess the influence of seasonal factors including constant exposure of the beekeepers to bee-related compounds such as allergens on the blood content composition, *in vitro* diagnostic test for allergy to bee venom were performed. The diagnostic tests conducted directly after a bee sting and after a minimum of 6 weeks after the sting showed no significant differences in the levels of total IgE antibodies, honeybee venom specific IgE antibodies, phospholipase A_2_ (the main allergen of honeybee venom) specific IgE antibodies, serum tryptase and honeybee venom specific IgG4 specific antibodies. These results are available on request.

**Table 2:** The concentrations of determined amino acids (LC-ESI-QqQ-MS/MS method) in serum of stung and not stung beekeepers (n = 27).

Amino Acid	Abbreviation	LOQ (µM)	Calculated concentration (µM) in real samples					
			Stung			Not stung		
			Min.	Max.	Median	Min.	Max.	Median
Taurine	Tau	0.5	26.5	173.6	71.7	33.4	134.6	76.3
L-Asparagine	Asn	0.5	19.7	57.2	37.4	28.9	69.0	43.6
L-Serine	Ser	0.5	54.0	150.3	98.6	74.7	174.0	118.1
Glycine	Gly	1.0	136.5	285.5	182.7	122.5	434.5	222.2
Hydroxy-L-proline	Hyp	0.2	5.1	45.5	11.0	4.3	37.8	12.6
Ethanolamine	EtN	0.5	4.3	11.1	6.8	6.3	11.4	7.7
L-Glutamine	Gln	0.5	175.3	461.1	339.9	364.5	569.0	440.1
L-Aspartic acid	Asp	0.1	4.3	23.5	10.8	5.4	20.4	11.2
L-Citrulline	Cit	0.5	11.4	35.9	22.6	12.4	40.0	25.7
L-Threonine	Thr	0.2	46.9	138.2	84.2	46.8	158.8	97.3
Sarcosine	Sar	0.2	0.9	3.0	1.8	0.8	3.1	1.7
β-Alanine	bAla	0.5	11.6	60.0	24.6	12.0	47.4	22.3
L-Alanine	Ala	0.2	213.4	620.3	387.4	308.1	661.2	472.3
L-Glutamic acid	Glu	0.5	47.2	205.4	92.1	33.0	104.2	62.2
L-Histidine	His	0.5	30.9	85.6	62.6	55.2	86.2	67.4
1-Methyl-L-histidine	1MHis	0.2	0.4	18.2	3.6	0.3	16.9	2.9
3-Methyl-L-histidine	3MHis	0.2	2.0	11.6	5.1	2.3	7.7	5.4
L-α-Aminoadipic acid	Aad	0.2	0.3	2.2	0.9	0.4	2.6	1.2
γ-Amino-n-butyric acid	GABA	0.05	0.1	0.6	0.3	0.1	0.7	0.3
D, L-β-Aminoisobutyric acid	bAib	0.2	0.6	4.2	1.3	0.7	4.7	1.4
L-α-Amino-n-butyric acid	Abu	0.2	7.1	24.1	16.1	11.9	30.3	18.9
L-Arginine	Arg	0.5	34.4	143.3	86.1	61.9	138.3	103.7
L-Proline	Pro	0.1	81.7	512.5	190.2	99.6	394.8	219.2
L-Ornithine	Orn	0.5	36.3	119.3	62.6	42.2	130.9	75.3
L-Cystine	Cys	1.0	1.0	11.8	1.5	1.0	7.4	1.8
δ-Hydroxylysine	Hyl	0.5	2.4	13.5	9.2	1.6	13.7	8.9
L-Lysine	Lys	0.5	63.7	244.1	180.0	141.2	250.8	194.0
L-Methionine	Met	0.1	6.9	24.0	16.0	11.7	31.3	19.8
L-Valine	Val	0.2	92.1	311.3	215.3	160.7	380.8	227.4
L-Tyrosine	Tyr	0.5	18.6	76.8	46.0	35.9	76.3	52.6
L-Isoleucine	Ile	0.5	24.5	96.3	60.0	45.6	122.9	68.6
L-Leucine	Leu	0.5	41.6	145.4	97.3	83.6	179.9	111.8
L-Phenylanalanine	Phe	0.2	25.9	82.0	46.5	42.1	71.9	53.3
L-Tryptophan	Trp	0.1	24.4	56.6	36.1	28.1	61.0	39.2

Univariate analysis has shown statistically significant differences in the concentration of: L-glutamic acid (Glu), p<0.001; L-glutamine (Gln), p<0.001; 3-methyl-L-histidine (3MHis), p = 0.022 and L-methionine (Met), p = 0.024. Amino acids for which serum concentrations decreased directly after a sting were: Gln (median for the non-stung individuals: 440.1 µM; median stung: 339.9 µM; 92.6% of the individuals had a decreased level of Gln after a sting) and Met (median non-stung: 19.8 µM; median stung: 16.0 µM; 74.1% of the individuals had a decreased level of Met after a sting). Elevated concentration of Glu after a sting was observed (median non-stung: 62.2 µM; median stung: 92.1 µM; 88.9% of the individuals had an increased level of Glu after a sting). Although the statistical analysis showed that 3MHis serum concentration was also increased, the results need to be critically assessed because only 55.6% of the individuals had higher levels of this amino acid after a sting.

**Figure 2:**
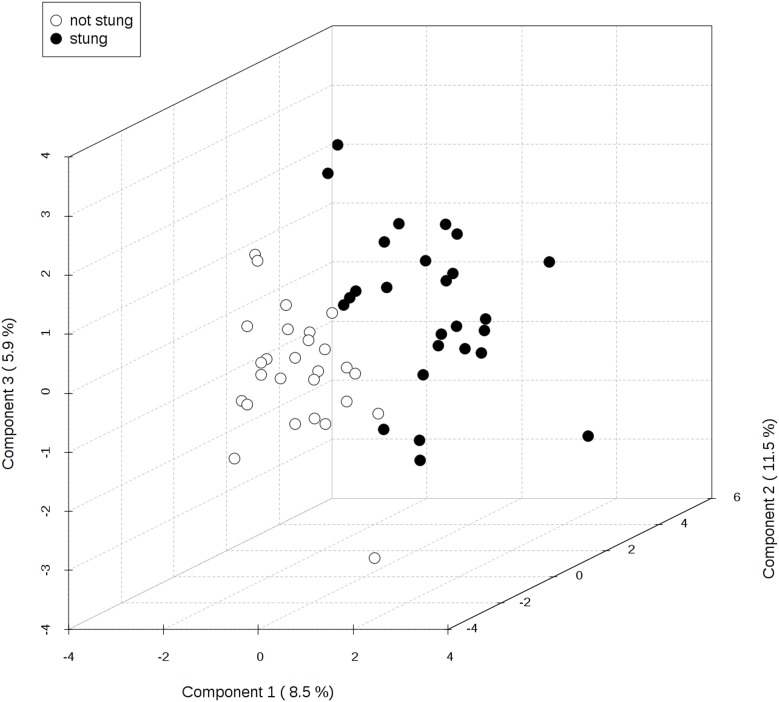
Three-dimensional (3D) partial least squares discriminant analysis separation using amino acids concentration-based metabolomics measurements in the serum of the beekeepers directly after a bee sting vs. the serum of the beekeepers after at least six weeks since the last sting (27 cases). The explained variances are shown in brackets.

**Figure 3:**
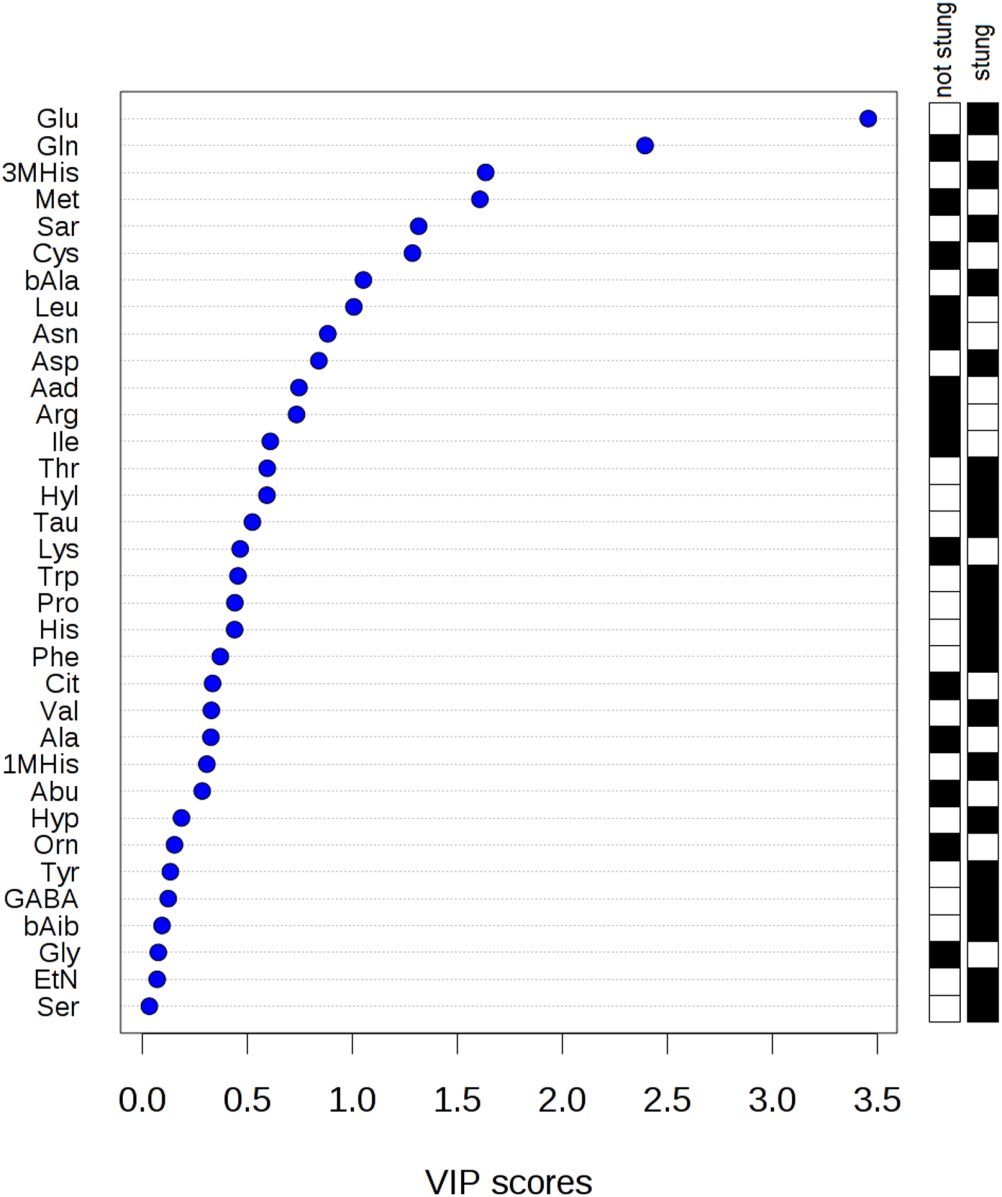
Variable importance in projection (VIP) plot: important features (analyzed serum free amino acids) identified by PLS-DA in a descending order of importance. The graph represents relative contribution of amino acids to the variance between the stung and non-stung individuals. High value of VIP score indicates great contribution of the amino acids to the group separation. The black and white boxes on the right indicate whether the metabolite concentration is increased (black) or decreased (white) in the serum of the stung vs. non-stung beekeepers.

Applying PLS-DA enabled us to distinguish the group of the stung and non-stung beekeepers (Figure 2). Clear clustering of 2 groups indicated that significant discrimination of the groups was achieved based on amino acid concentration differences. It was shown that the contrast between the two class assignment is statistically significant (p<0.001). The plot presented in Figure 3 ranks the analyzed amino acids according to the variable importance scores (VIP). PLS-DA method indicated the same amino acids as significant as the paired t-test. Taking VIP cut-off of around 1.5, the following amino acids were significant: Glu, Gln, 3MHis and Met. Only two features (Glu and Gln) had the VIP score higher than 2.0 that suggested the highest importance of these variables to the whole model.

**Figure 4:**
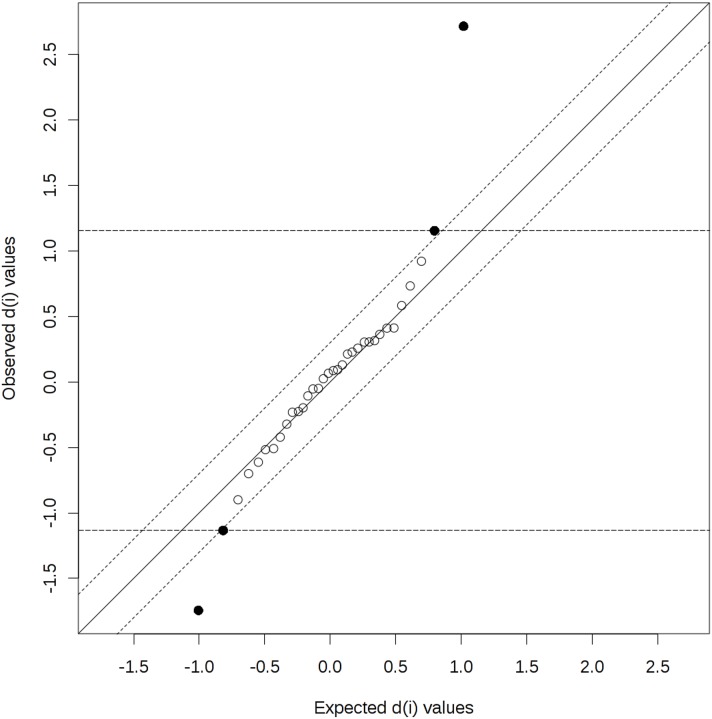
Significant features identified by SAM (Significance Analysis of Microarray (Delta = 0.3). The more the variable deviates from the “observed-expected d” line, the more likely it is to be significant. The bold dots represent features that exceed the specified threshold (cutlow = –1.134, cutup = 1.155). Significant positive compounds (i.e. mean concentration in the serum of the beekeepers directly after a bee sting > mean concentration in the serum of the beekeepers after at least six weeks since the last sting): Glu (d.value = 2.717), 3MHis (d.value = 1.155). Significant negative compounds (i.e. mean concentration in the serum of the beekeepers directly after a bee sting < mean concentration in the serum of the beekeepers after at least six weeks since the last sting): Gln (d.value = –1.744), Met (d.value = –1.134).

In the next step of the statistical evaluation, the Significance Analysis of Microarray (SAM) was used. A SAM plot for Delta = 0.3 displays a linear correlation between the expected and observed values determined for the studied amino acids (Figure 4). A total of 4 compounds were identified above the chosen threshold (Delta = 0.3) - Glu, Gln, 3MHis and Met. These results are in agreement with the results obtained using both the t-tests and PLS-DA.

**Figure 5:**
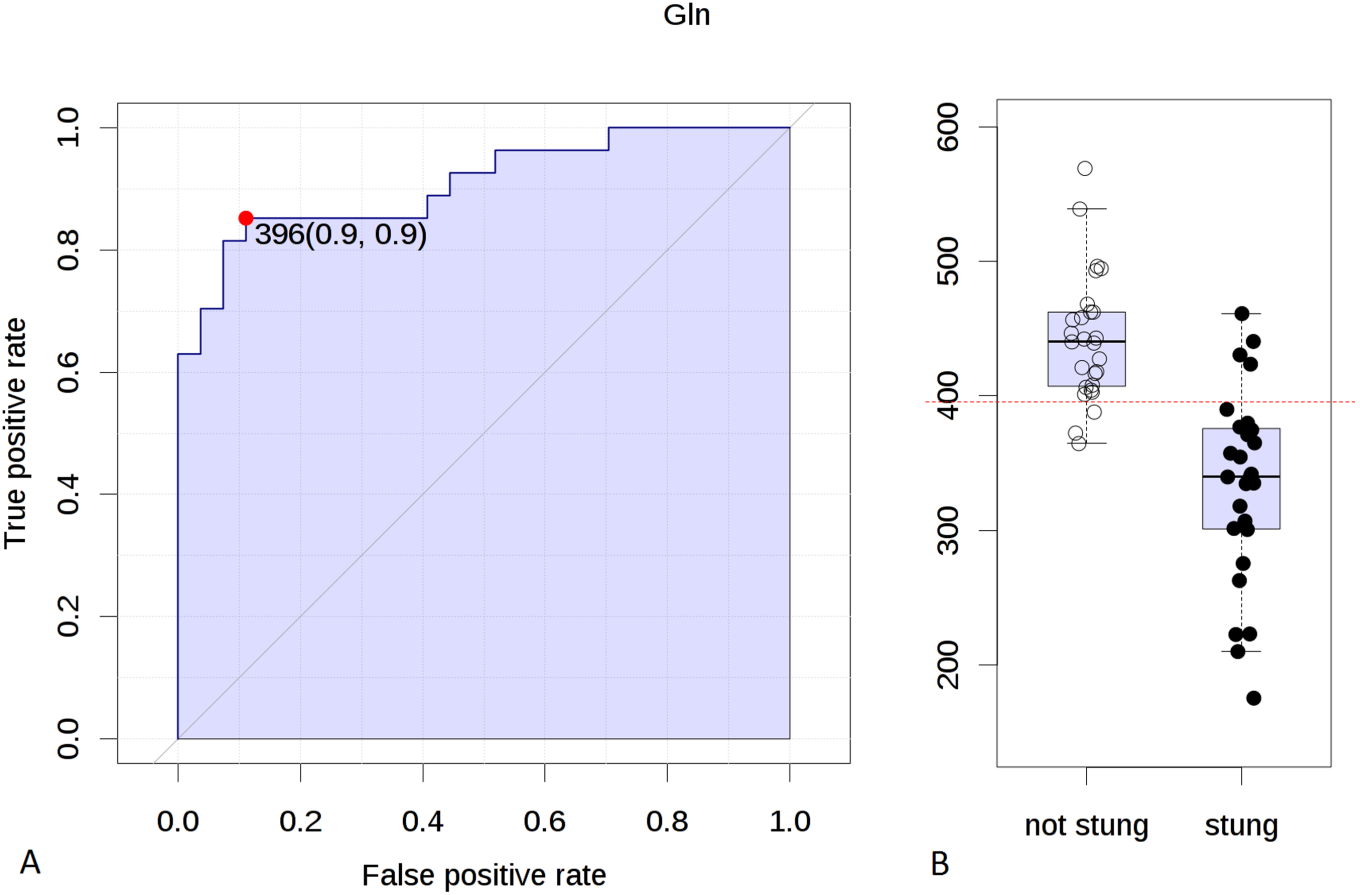
**A:** Receiver operating characteristic (ROC) curve for Gln. Cut-off value: 396.0 µM/l. Specificity: 90%, sensitivity: 90%. Area under the curve (AUC): 0.912, confidence interval: 95% (0.816–0.979); **B:** Concentration of Gln in two groups of beekeepers (directly after a sting and at least six weeks since the last sting). Dotted line indicates a cut-off value of 396.0 µM/l.

In order to determine a cut-off value of differentiating factors (concentrations of amino acids) for the stung and non-stung beekeepers, the receiver operating characteristic (ROC) curves were used. ROC curve for Gln indicated that the concentration value of 396.0 µM/l discriminated the stung and non-stung beekeepers with the specificity of 90% and sensitivity of 90% (Figure 5). The area under the curve (AUC) was 0.912 and considering the confidence interval (CI) of 95% it ranged from 0.816 to 0.979. The cut-off value calculated from the ROC curve for Glu was 80.8 µM/l (specificity = 80%, sensitivity = 60%, AUC = 0.775, 95% CI: 0.63–0.879).

Apart from LC-ESI-QqQ-MS/MS analyses of the serum specimens, some additional experiments were carried out to confirm the data obtained.

The limit of quantitation (LOQ) of the method for all amino acids was ≤1 µM (Table 3). The linear dynamic range was from LOQ value up to ≥10 000 µM for all analyzed amino acids (this resulted in at least 4 orders of magnitude).

**Table 3:** Validation parameters of the LC-ESI-QqQ-MS/MS method used for determination of amino acids in serum of beekeepers.

Amin Aicd	Analyte Q1/Q3 Mass (amu)	IS Q1/Q3 Mass (amu)	Precision study R.S.D. (%) (n = 9)						Control plasma	
			Concentration		Analyte RT		IS RT			
			Intraday precision	Interday precision	Intraday precision	Interday precision	Intraday precision	Interday precision	Reference Range (µM)	Calc. conc. (µM)
Tau	274.1/121.1	266.1/113.1	6.54	2.21	0.66	1.87	0.51	2.33	25.9–38.8	33.9
Asn	281.2/121.1	273.1/113.1	2.96	6.04	0.44	2.38	0.53	1.67	26.4–39.7	32.6
Ser	254.2/121.1	246.2/113.1	1.32	10.35	0.43	2.64	0.48	1.87	28.6–100.3	86.5
Gly	224.1/121.1	216.1/113.1	3.10	10.05	0.41	2.28	0.39	2.37	137.0–206.0	193.3
Hyp	280.1/121.1	272.1/113.1	2.10	10.99	0.46	2.66	0.41	2.54	-	-
EtN	210.2/121.1	202.2/113.1	2.29	7.72	0.32	2.17	0.32	2.28	-	-
Gln	295.2/121.1	287.2/113.1	1.59	11.49	0.28	2.18	0.39	2.74	220.0–330.0	253.4
Asp	282.1/121.1	274.1/113.1	6.03	7.05	0.31	2.56	0.21	2.07	10.2–15.3	11.5
Cit	324.2/121.1	316.2/113.1	8.23	18.24	0.19	1.98	0.25	2.27	16.8–25.2	20.2
Thr	268.2/121.1	260.2/113.1	1.46	10.23	0.24	2.20	0.24	2.38	79.8–120.0	98.2
Sar	238.2/121.1	230.2/113.1	6.06	2.86	0.26	2.40	0.35	1.90	-	-
bAla	238.2/121.1	230.2/113.1	2.81	8.34	0.27	2.08	0.16	2.20	-	-
Ala	238.2/121.1	230.2/113.1	3.62	5.92	0.15	2.03	0.09	2.40	279.0–418.0	408.1
Glu	296.2/121.1	288.2/113.1	4.77	6.74	0.18	2.04	0.15	1.99	147.0–221.0	162.8
His	304.2/121.1	296.2/113.1	6.89	3.26	0.25	3.09	0.19	1.96	57.8–86.7	68.7
1MHis	318.2/121.1	310.2/113.1	0.58	6.53	0.25	2.65	0.25	2.03	-	-
3MHis	318.2/121.1	310.2/113.1	3.57	9.07	0.15	2.18	0.30	3.09	-	-
Aad	310.2/121.1	302.2/113.1	11.54	11.16	0.00	1.48	0.00	2.65	-	-
GABA	252.2/121.1	244.2/113.1	10.76	27.15	0.08	1.13	0.00	2.39	-	-
bAib	252.2/121.1	244.2/113.1	7.01	6.28	0.07	1.37	0.00	2.62	-	-
Abu	252.2/121.1	244.2/113.1	2.70	10.00	0.07	1.40	0.07	1.39	10.9–16.4	16.1
Arg	323.2/121.1	315.2/113.1	7.82	6.02	0.15	2.11	0.13	1.84	45.3–67.9	54.0
Pro	264.2/121.1	256.2/113.1	3.27	8.39	0.07	1.45	0.07	2.05	159.0–238.0	230.5
Orn	429.3/121.1	413.3/113.1	1.29	10.10	0.07	1.63	0.00	1.48	56.1–84.1	56.7
Cys	537.2/121.1	521.2/113.1	23.83	20.05	0.07	1.29	0.07	1.48	-	-
Hyl	459.3/121.1	443.3/113.1	5.23	8.58	0.08	1.74	0.39	1.44	-	-
Lys	443.3/121.1	427.3/113.1	4.73	12.09	0.07	1.29	0.07	1.40	92.1–138.0	129.9
Met	298.2/121.1	290.2/113.1	2.38	10.20	0.11	1.07	0.17	2.11	41.9–62.8	47.3
Val	266.2/121.1	258.2/113.1	1.28	10.59	0.17	1.00	0.17	1.38	145.0–217.0	197.2
Tyr	330.2/121.1	322.2/113.1	6.13	10.82	0.11	0.76	0.16	1.56	39.2–58.7	43.9
Ile	280.2/121.1	272.2/113.1	2.74	7.01	0.00	0.97	0.00	1.41	46.1–69.2	55.1
Leu	280.2/121.1	272.2/113.1	0.73	9.05	0.00	1.09	0.00	1.34	127.0–191.0	156.1
Phe	314.2/121.1	306.2/113.1	1.22	8.16	0.00	0.94	0.00	2.27	59.3–88.9	65.0
Trp	353.2/121.1	345.2/113.1	1.38	6.98	0.48	0.49	0.48	1.29	39.2–58.7	39.6

Labeling efficiency was checked by using non-proteogenic amino acids (norleucine and norvaline). The workflow efficiency is acceptable when the amount of those amino acids recovered is within 20% of the value listed on the Certificate of Analysis supplied by AB Sciex with each aTRAQ kit. All of the measured norleucine and norvaline concentrations were within that range. Accuracy of the method was examined by analyzing the Control Plasma which contained 25 amino acids. All of the measured amino acid concentrations were in agreement with the reference ranges established for this reference material. The Control Plasma was analyzed with each new set of aTRAQ reagents. Results of one of the Control Plasma analysis is shown in Table 3. Analyses of the Control Plasma also proved a satisfactory performance of the entire methodology.

To verify the precision of the applied method, relative standard deviation (RSD) for amino acid concentrations and retention times of analytes and internal standards were evaluated. As can be seen in Table 3, very low RSD values for the retention time of the analyzed compounds were obtained, and they were below 1% and 3% for inter-day and intra-day precision, respectively. The determined RSD values proved a good precision of the applied method.

Metabolomics is known to be a powerful tool in elucidating the mechanisms of complex disorders or explaining response of the organism to physiological or pathological stimuli. A review of the Human Metabolome Database (www.hmdb.ca) was performed to determine the role of some amino acids in response of the human body to a honeybee sting. Glutamic acid is described in the literature as the most abundant fast excitatory neurotransmitter [29], [30]. It is also involved in energy processes by entering the Krebs cycle and in GABA biosynthesis, which is an inhibitory mediator in the brain. High levels of glutamic acid are connected with headaches and other neurological conditions [31], [32]. Higher levels of glutamic acid in the serum of the beekeepers directly after a bee sting are probably connected with the stress reaction of the body and stimulation of the nervous system. On the other hand, a decreased concentration of glutamine directly after a sting may be due to the fact that glutamic acid can be generated from glutamine with the concomitant production of ammonia in a process mediated by glutaminase. Moreover, a decrease in glutamine level can be explained by impaired buffering capacity of the body due to the activity of the honeybee venom. It is known that glutamine is an amino acid that serves as a buffer to the biological fluids and a nitrogen carrier [33]. Consequently, this results in detoxifying process, because nitrogen from amino acids metabolism is funneled into the urea cycle in the liver. Thus, the decrease in glutamine concentration in the serum of the beekeepers directly after a bee sting may suggest its participation in the detoxification of the bee venom spreading throughout the body.

## Concluding Remarks

This study showed that the implementation of a complex analytic-bioinformatic-clinical strategy based on up-to-date achievements of mass spectrometry may allow to discriminate the individuals directly after a sting and individuals after a minimum of 6 weeks following the last sting. Mass spectrometry-based serum metabolite profiling is a promising technique to analyze complex metabolic alterations, which may broaden the understanding of the human body response to a honeybee sting. Thus, determination of serum AAs sheds a new light on the metabolic basis of the reaction to *Hymenoptera* sting.

## Supporting Information

File S1 Raw data of the concentrations of determined amino acids (LC-ESI-QqQ-MS/MS method) in serum of stung and not stung beekeepers (n = 27).
(XLSX)
